# SENIOR COMMUNITY CENTER AS A SOCIAL ENGAGEMENT PLATFORM FOR OLDER ADULTS IN NEPAL: AN ADAPTATION OF WESTERN CONCEPT

**DOI:** 10.1093/geroni/igz038.3548

**Published:** 2019-11-08

**Authors:** Sabita Shrestha, Tina Colson

**Affiliations:** 1 Southeastern Louisiana University, Hammond, Louisiana, United States; 2 Southern Illinois University, Carbondale, Illinois, United States

## Abstract

Older adults around the world are living longer. Similarly, in Asian countries longevity of older adults have significantly altered the demographics shift as well as the cultural landscape and needs of the society. These changes have compounded with challenges and needs as a community grapples with how to best take care of aging population. Nepal, a developing country, is also faced with a similar demographic shift among the geriatric population. This shift has brought challenges and needs to communities such as health care, daily living needs, social support systems, economic needs, etc. The geriatric population will require social support systems as they age. Historically, older adults have relied on traditional family support systems for their care and needs maintaining cultural values and norms which may burden immediate or extended family members. Recently, traditional family structures along with social support systems are breaking away from their family trees due to community advancement and modernization, and many are leaving for better economic opportunities. This trend has left many older adults alone in social isolation. Despite challenges in the community, Nepal government doesn’t offer infrastructure for social engagement for older adults. One solution to prevent isolation and loneliness is to establish “senior community centers” (western based concept) for social engaging older adults. Based on ecological framework, this presentation proposes a need of “Senior Community Centers” for older adults where they can become involved in social engagements and receive social supports outside traditional family support systems; thus, optimizing their health and well-being.

